# The impact of thyroid hormone concentration fluctuations on colon cancer proliferation and growth

**DOI:** 10.3389/fendo.2025.1576665

**Published:** 2025-07-17

**Authors:** Fang Ren, Yaqi Zhang, Cancan Hui, Zekai Li, Datong Deng

**Affiliations:** ^1^ The General Practice Department, The First Affiliated Hospital of Anhui Medical University, Anhui Medical University, Hefei, China; ^2^ The Endocrinology and Metabolism Department, The First Affiliated Hospital of Anhui Medical University, Anhui Medical University, Hefei, China; ^3^ The Geriatric Endocrinology Department, The First Affiliated Hospital of Anhui Medical University, Anhui Medical University, Hefei, China; ^4^ Institute of Endocrinology and Metabolism, Anhui Medical University, Hefei, China

**Keywords:** thyroid hormone, hypothyroidism, colon cancer, TUNEL, hematoxylin eosin staining

## Abstract

**Background:**

To examine the effects of thyroid hormone (TH) levels on colon cancer progression, HCT-116 colon cancer cells were inoculated into the axillary region of thymus-deficient male BALB/c nude mice. Mild hypothyroidism and hyperthyroidism were then induced to observe tumor growth patterns under different TH conditions.

**Methods:**

Following subcutaneous tumor implantation, mice were randomly divided into three groups: hyperthyroid (levothyroxine-treated), hypothyroid (methimazole-treated), and control (saline-treated). Tumor volume and final mass were monitored throughout the study period. Excised tumors were subjected to histological analysis including hematoxylin-eosin (HE) staining, immunofluorescence, and TUNEL assay for apoptosis detection.

**Results:**

In the tumor-bearing experiment conducted with nude mice, the growth curve and tumor weight of the methimazole group exhibited a inhibitory trend compared to the saline group (P<0.05). In the immunofluorescence staining experiment, Ki67 expression was higher in control and hyperthyroid groups than hypothyroid (P<0.01 and P<0.001, respectively), with no significant control-hyperthyroid difference (P>0.05). TUNEL staining results demonstrated no significant presence of TUNEL-positive cells in the subcutaneous tumor tissue of the control group. Additionally, the proportion of TUNEL-positive cells in the subcutaneous tumor tissue of the levothyroxine group was slightly lower than in the control group. (P>0.05), whereas the proportion of TUNEL-positive cells in the methimazole group increased significantly (P<0.0001).

**Conclusion:**

Fluctuations in thyroid hormone levels in the body can affect the proliferation and growth of colon cancer cells. Furthermore, reducing thyroid hormone levels can inhibit the proliferation of colon cancer cells while promoting their apoptosis.

## Introduction

The thyroid gland secretes two main thyroid hormones: thyroxine (T_4_) and triiodothyronine (T_3_). Among them, T_3_ is the primary hormone responsible for exerting physiological effects and serving as a key indicator of the body’s metabolic state (1). Thyroid hormones are widely distributed in most tissues and can regulate the expression of target genes in different tissues, including cellular metabolic rate, cardiovascular and digestive functions, muscle development and activity, brain development, and bone turnover. They are the main regulatory factors for vertebrate development and physiological functions (2). When present in the body, thyroid hormones can enhance the cellular oxidation rate, generate heat, accelerate the metabolism of sugars, fats, and proteins, and promote growth and development (3). In recent years, various molecular pathways through which thyroid hormones act on tumor cells have been elucidated (4). As a product of gene mutations in normal cells, tumor cells continue to rely on thyroid hormones for regulation during their proliferation and growth (5–7). A study has shown that the use of levothyroxine increases the risk of cancer (8). However, a recent single-center analysis suggests that a history of hypothyroidism and levothyroxine thyroid replacement therapy may be associated with beneficial outcomes in patients with resectable cancer (9).

Colon cancer most frequently manifests at the juncture where the sigmoid colon and rectum converge, ranking third among gastrointestinal tumors. The accelerating processes of urbanization and industrialization have led to an accumulation of risk factors, including unhealthy dietary habits and increasing environmental pollution. Consequently, the incidence rate of colon cancer has remained consistently high, with an annual upward trend observed. Furthermore, the age demographic of affected patients has gradually shifted towards younger age groups (10, 11).

The relationship between the proliferation and growth of tumor cells and thyroid hormones warrants further investigation. This experiment utilizes colon cancer cells to examine how variations in thyroid hormone levels affect cancer cell proliferation and growth. Meticulously modulating the concentration of thyroid hormones within the body may impede tumor progression or induce apoptosis.

## Materials and methods

### Experimental cells

The HCT116 human colorectal cancer cell line was obtained from Pricella Biotechnology Co., Ltd. and stored in a liquid nitrogen tank at the Endocrine Molecular Laboratory of the First Affiliated Hospital of Anhui Medical University.

### Animals

Male BALB/c mice, aged 4–5 weeks and weighing 15–18 grams, were purchased from the Experimental Animal Centre of Anhui Medical University. The mice should be housed in a specific pathogen-free environment with a temperature of 20-25°C and be allowed a one-week acclimatization period.

### Main reagents

Methylimidazole (10 mg, Jiangsu Merck Pharmaceutical Co., Ltd.), levothyroxine sodium tablets (25 μg, Shenzhen Zhonglian Pharmaceutical Co., Ltd.), Ki67 (Affinity); and sheep anti-rabbit secondary antibody (Wuhan Sanying).

### Main instruments

The following equipment was used for this study: an ELISA detector (Biotek), a TUNEL apoptosis detection kit (Jiangsu Kaiji), a 37°C incubator (Jinghong Experimental Equipment Co., Ltd.), a paraffin ultra-thin slicer (Shanghai Leica Instrument Co., Ltd.), a paraffin embedding machine (Hanjunjie Electronics Co., Ltd.) and a light microscope (Nikon, Japan).

### Preparation of tumor bearing mice

A total of 18 male nude mice were randomly assigned to three groups of six: a control group, a levothyroxine group, and a methimazole group (12, 13). The tumor mass was measured in preliminary experiments (n = 3 per group), and the t-test indicated that 6 mice per group were required for the formal experiment. HCT-116 cells were cultured in a 75 cm² culture bottle and cells in the logarithmic growth phase were selected for digestion to ensure a high cell survival rate. The digested cells were resuspended in nutrient solution and 10 µl was immediately added to a centrifuge tube containing 90 µl. The tube was shaken and mixed well and 10 µl was added to a cell counting plate for counting. Each mouse was inoculated with 100 µl of the suspension to ensure a cell count of approximately 1×10^6^. When inoculating cancer cells, the injection site must be fixed uniformly, a unified unit syringe must be used to maintain the same volume, and all operations must be carried out by the same person in batches. Consistent feeding conditions must also be maintained to reduce the impact of environmental fluctuations. After tumor sizes reached 50–100 mm³, medication was administered.

### *In vivo* anti-tumor activity assay

The levothyroxine group and the methimazole group received daily intraperitoneal injections of 4 mg/kg (14) methimazole and 50 µg/kg levothyroxine sodium (based on the drug’s effective concentration, obtained by converting body surface area). The control group received the same volume of saline for 20 days.

### Measurement of serum thyroid hormone

Approximately 1 ml of blood was collected from the orbital vein of the male mouse prior to euthanasia, when the tumor had reached a certain size. The serum was then separated by high-speed centrifugation. An enzyme-linked immunosorbent assay (ELISA) was then used to determine the levels of T_3_, T_4_ and TSH in the serum.

### Hematoxylin and eosin staining experiment

Fresh tumor tissue should be fixed in 4% paraformaldehyde for at least 24 hours, then dehydrated in a graded ethanol series, embedded in paraffin wax, sectioned, and stored at room temperature. The slices are then placed in xylene for dewaxing, followed by rehydration in a graded ethanol series. They were then placed in a hematoxylin staining bath and stained for 5 minutes. The hematoxylin solution was then rinsed off with running water, followed by 1% hydrochloric acid ethanol for 2 seconds. The slices were rinsed in running water until they turned blue, which took about 10 minutes, and then stained with a 0.5% eosin solution for 2 minutes. Dehydrate again using the gradient ethanol, clear using xylene, seal using neutral gum, and then leave to air dry in a ventilated area. Observe and photograph under a light microscope.

### Immunofluorescence staining experiment

The slices were dissolved in xylene for dewaxing and in a gradient of ethanol for hydration. Antigen repair was performed by boiling the slices for 20 minutes on a low heat. The slices were then left to cool naturally. The slices were then placed in a 3% H_2_O_2_ solution and incubated at room temperature for 10 minutes to block endogenous peroxidase. After cleaning and drying, the slices were blocked with 5% BSA for 20 minutes. After removing the BSA solution, the slices were incubated with the Ki-67 primary antibody at 4°C overnight. Next, fluorescent secondary antibodies of the corresponding species were added to the slices and incubated at room temperature for 50 minutes. After cleaning, the slices were stained with DAPI and sealed with glycerol. Finally, the slices were visualized and photographed under an inverted immunofluorescence microscope.

### *In situ* notch end marking experiment

The slices were dissolved in a proteinase working solution and reacted at 37°C for 20 minutes. A 3% H_2_O_2_ solution was then used to block endogenous peroxidase. The slices were then incubated in TDT enzyme buffer at 37°C for one hour and washed three times with PBS. The slices were then labelled with an HRP working solution at 37°C for 30 minutes. Except for the PBS solution, 50–100 µl of freshly prepared diaminobenzidine (DAB) solution was added to each slice. It was then allowed to react at room temperature until the depth of brown could be determined under the microscope. Development was then stopped with PBS. The slices were stained with hematoxylin for 25 seconds and rinsed under running water for three to five minutes to return to blue. The slices were then dehydrated in a graded alcohol series, cleared in xylene, and sealed with neutral gum.

### Statistics

All statistical analyses were conducted using GraphPad Prism version 10.0 and the data were presented as means with their respective standard deviations (SD). One-way or two-way analysis of variance (ANOVA) was used for data comparisons among multiple groups, while comparisons between groups were performed using t-test. A P-value of less than 0.05 indicates a statistically significant difference.

## Results

### Comparison of thyroid hormone levels

**Figure 1:**
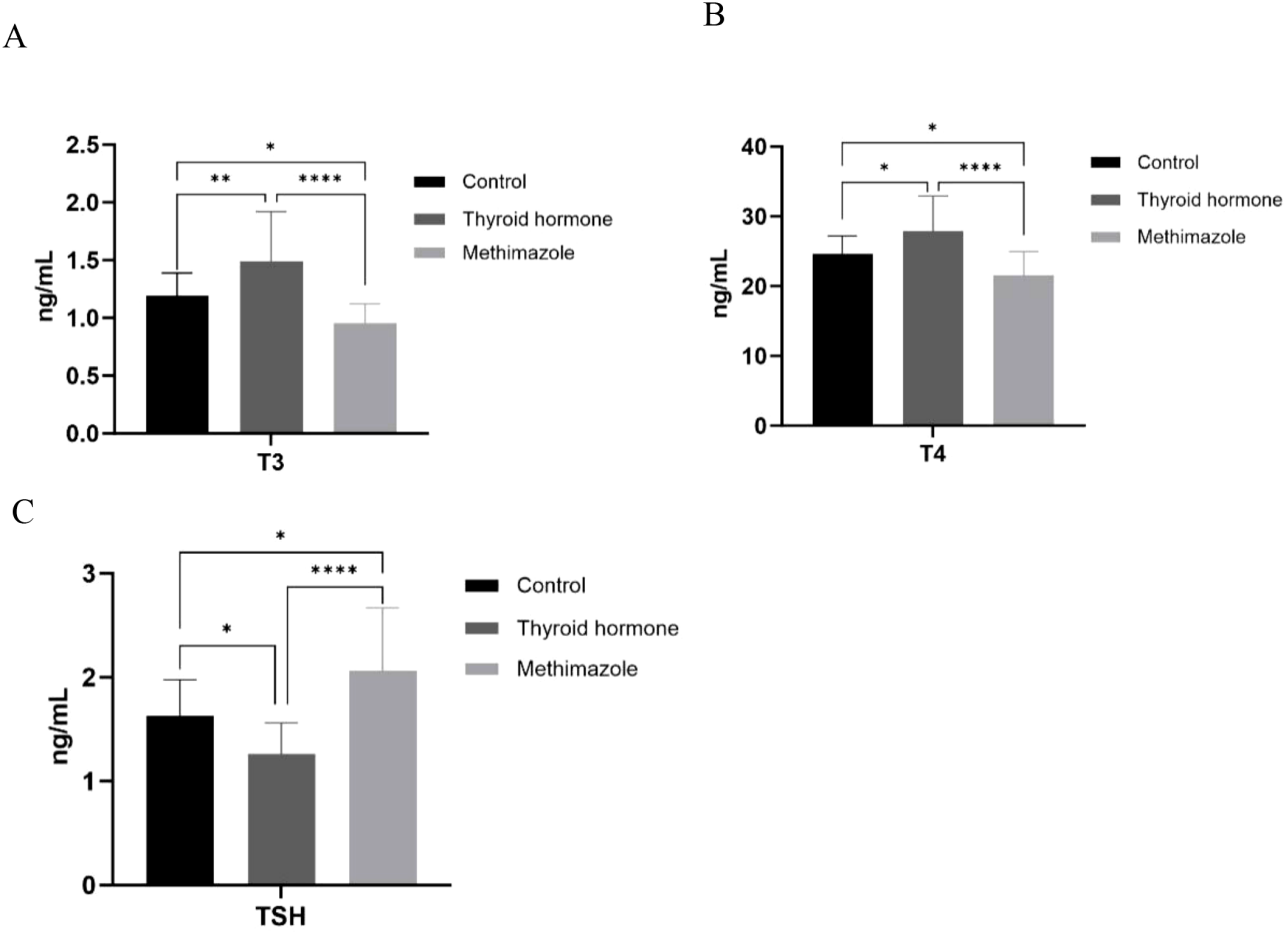
**(A–C)** Comparison of T3, T4, and TSH content among three groups. n=6 for each group, *P<0.05, **P<0.01, ****<0.0001. (Data were analyzed for statistically significant differences using one-way ANOVA).

The ELISA test results indicated that T_3_ and T_4_ levels increased and TSH levels decreased in the levothyroxine group. Conversely, the levels of T_3_ and T_4_ decreased in the methimazole group, while the TSH level increased. Both of these outcomes are statistically significant, indicating the successful simulation of hyperthyroidism and hypothyroidism in nude mice through the changes in thyroid hormone levels (*P<0.05, **P<0.01, ****P<0.0001, as shown in **Figure 1**).

### Tumor growth trend

**Figure 2:**
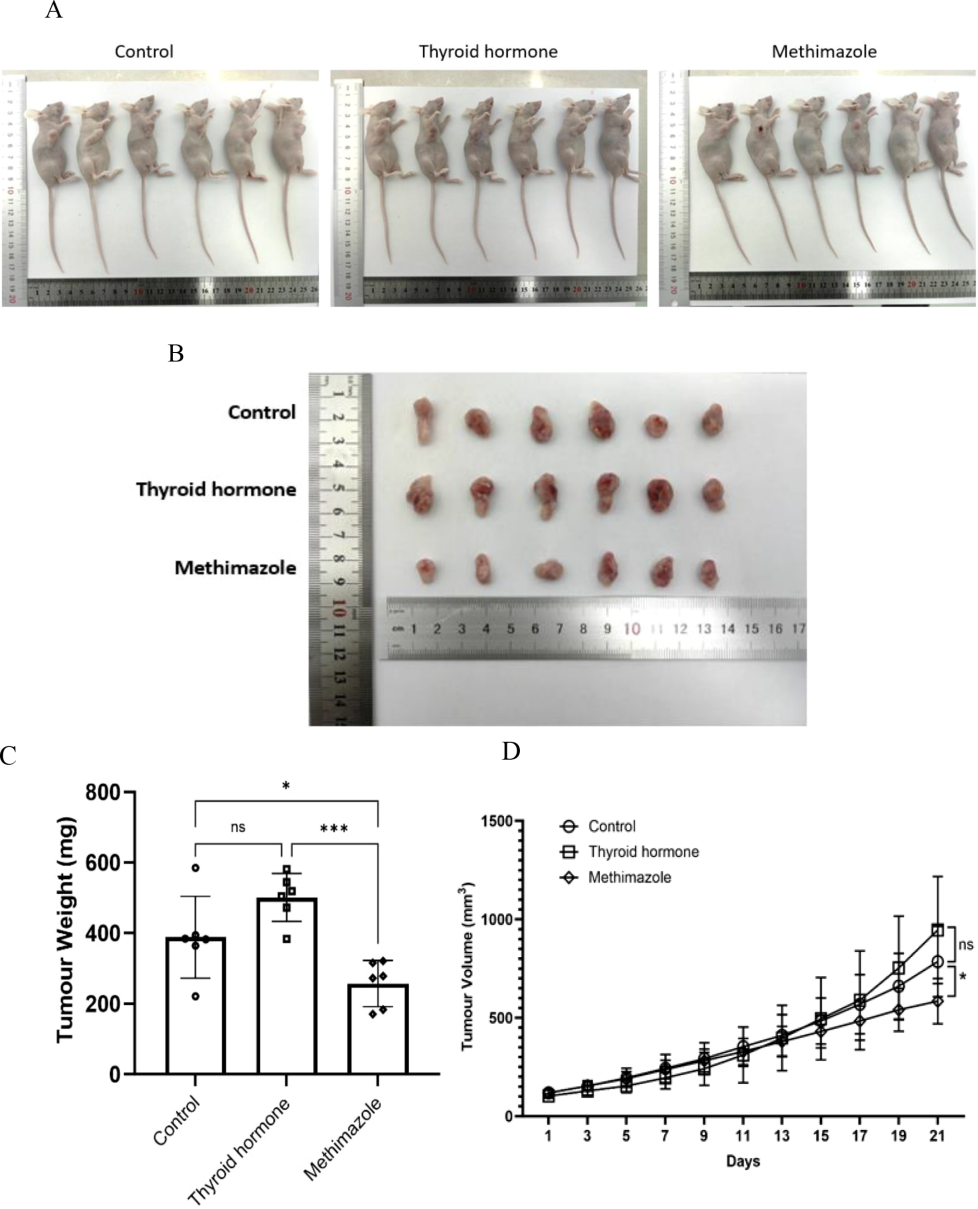
Comparison of tumor quality and trend of tumor volume growth among different groups. **(A, B)** Tumor images. **(C)** Tumor weight at the end of the experiment, n=6 for each group (Data were analyzed for statistically significant differences using one-way ANOVA, *P<0.5, ***P<0.001). **(D)** Three groups of tumor volume growth trends. Record changes in tumor volume every 2 days throughout the entire treatment period. n=6 for each group. (Data were analyzed for statistically significant differences using two-way ANOVA, ns, no statistical significance, *P<0.05).

After the experiment is completed, the nude mice will be euthanized and corresponding group photos will be taken. Then, the tumor bodies of the three groups of nude mice will be removed for final volume and weight comparison. (as shown in **Figure 2A, B**). The results indicated that the tumor weight and growth curve of the methimazole group showed an inhibitory trend compared to the control group (P < 0.05; see **Figures 2C, D**). Conversely, although the tumor volume and weight of the levothyroxine group increased compared to the saline group, this difference was not statistically significant (P>0.05, see **Figures 2C, D**).

### Morphological analysis of tumor tissue

**Figure 3:**
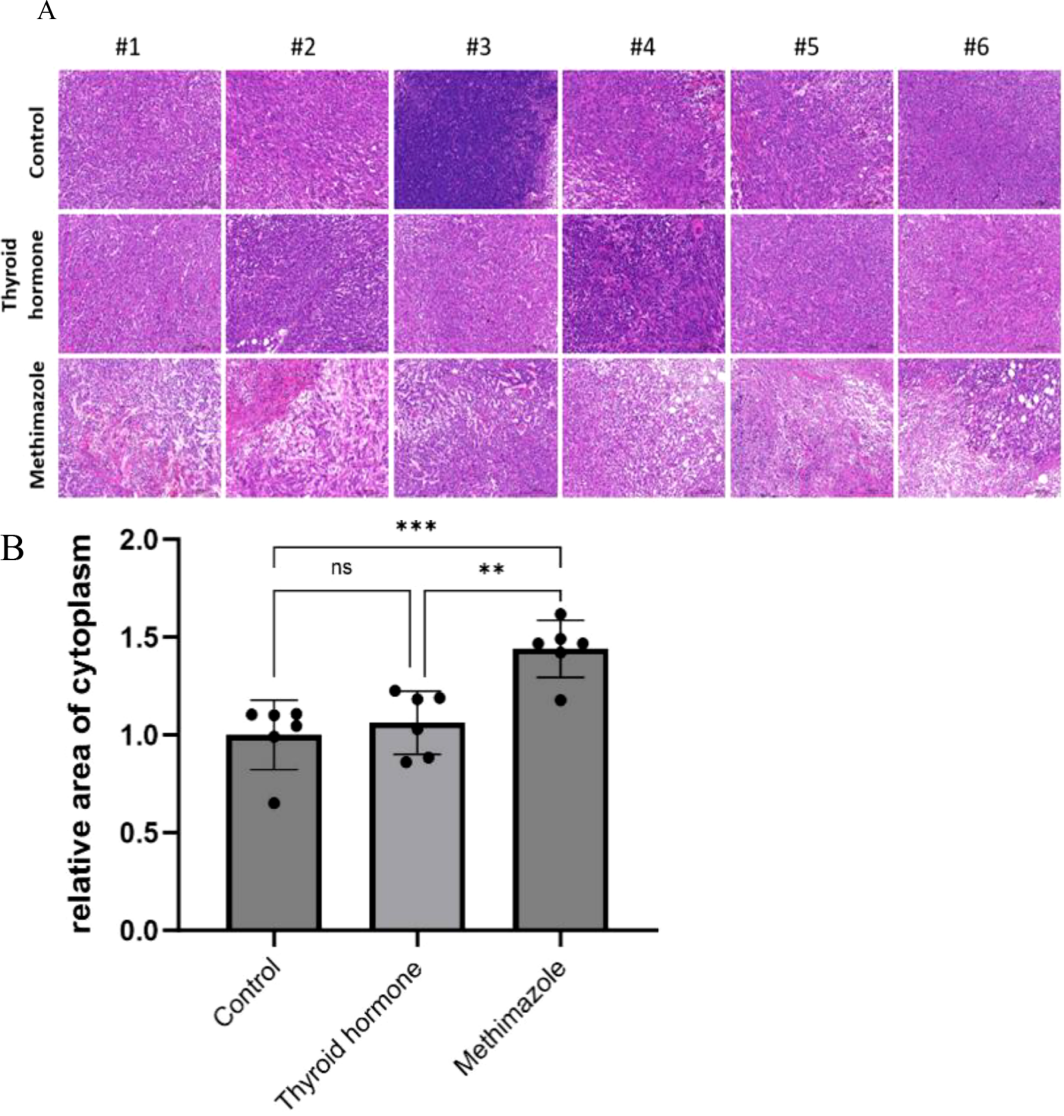
Comparison of cell morphology among three groups stained with HE. **(A)** Pathological images, Scale bar:200um. **(B)** Relative area of cytoplasm, n=6 for each group. (Data were analyzed for statistically significant differences using one-way. ns, no statistical significance, **P< 0.01 ***P< 0.001).

Histological analysis via HE staining revealed distinct structural characteristics among the different experimental groups. Following HE staining, the nucleus appears dark blue or purple, while the cytoplasm appears pink. In the saline group, the subcutaneous tumor tissue had a dense structure with no significant structural lesions and the tissue cell morphology remained intact. Similarly, the subcutaneous tumor tissue in the levothyroxine group had a comparable dense structure to that of the control group and no evident pathological changes were observed in either group. In stark contrast, the subcutaneous tumor tissue in the methimazole group exhibited a loose structure with disrupted cell morphology, indicating clear signs of disintegration (as illustrated in **Figure 3A**). (as illustrated in **Figure 3A**). The relative area stained pink represents the relative area of the cytoplasm and provides an objective standard for the degree of cell lysis (as demonstrated in **Figure 3B**).

**Figure 4:**
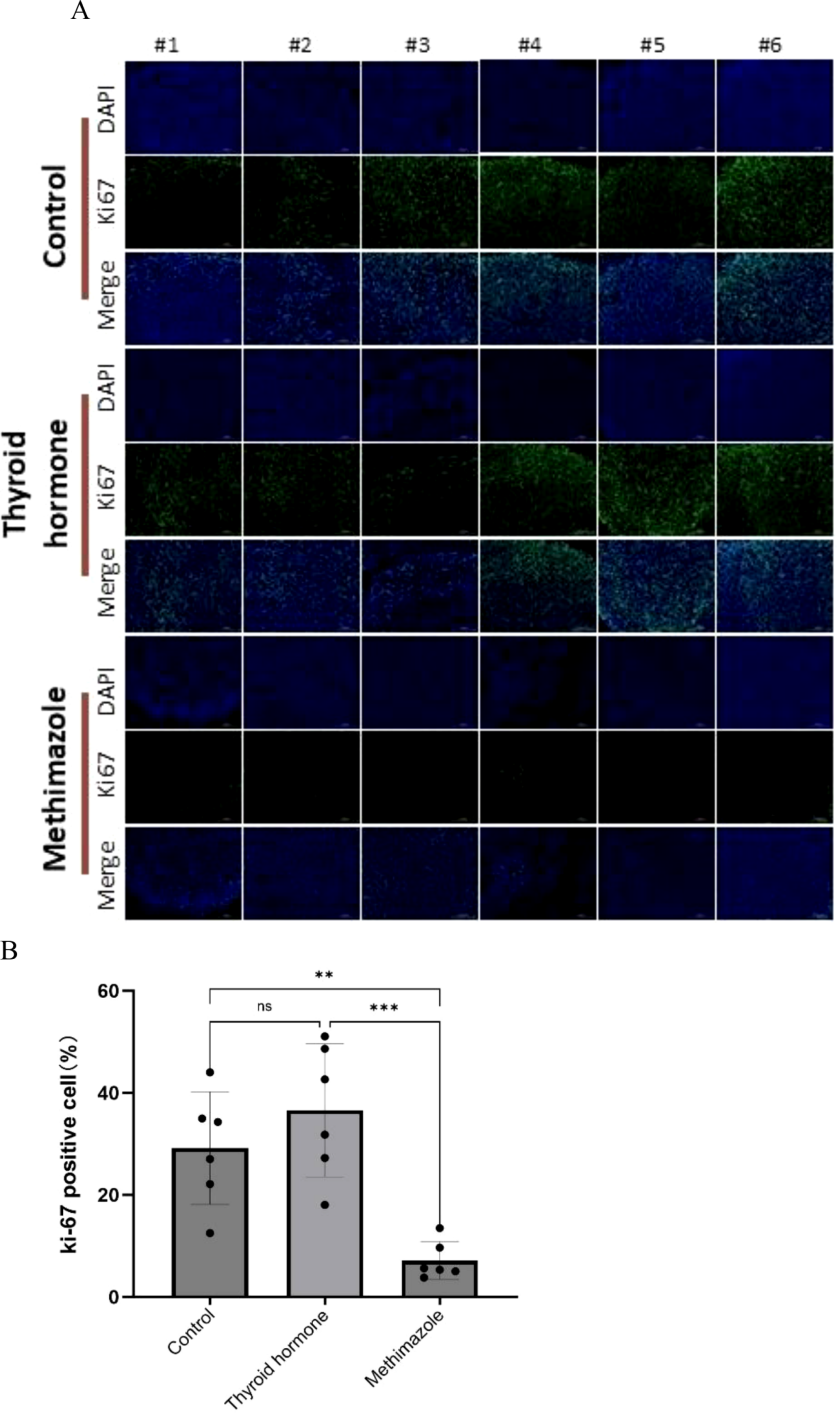
Comparison of three groups of immunofluorescence staining. **(A)** Pathological images, Scale bar:200um. **(B)** Proportion of Ki67 positive cells, n=6 for each group. (Data were analyzed for statistically significant differences using one-way. ns, no statistical significance, **P< 0.01 ***P< 0.001).

In the context of tissue fluorescence staining, the blue DAPI label highlights the nucleus, while the green label denotes Ki67 protein staining, which is expressed within the nucleus. A clear observation revealed that the methimazole group exhibited a significantly smaller area of green fluorescence compared to the saline and levothyroxine groups (as illustrated in **Figure 4A**). The results of tissue fluorescence staining indicate that the expression level of Ki67 protein in the subcutaneous tumor tissues of the control group and the levothyroxine group is higher than that of the methimazole group (P<0.01and P<0.001, respectively), as shown in **Figure 4B**. Although the Ki67 fluorescence positivity rate in the levothyroxine group showed a marginal increase compared to the control group, this difference did not achieve statistical significance (P>0.05).

**Figure 5:**
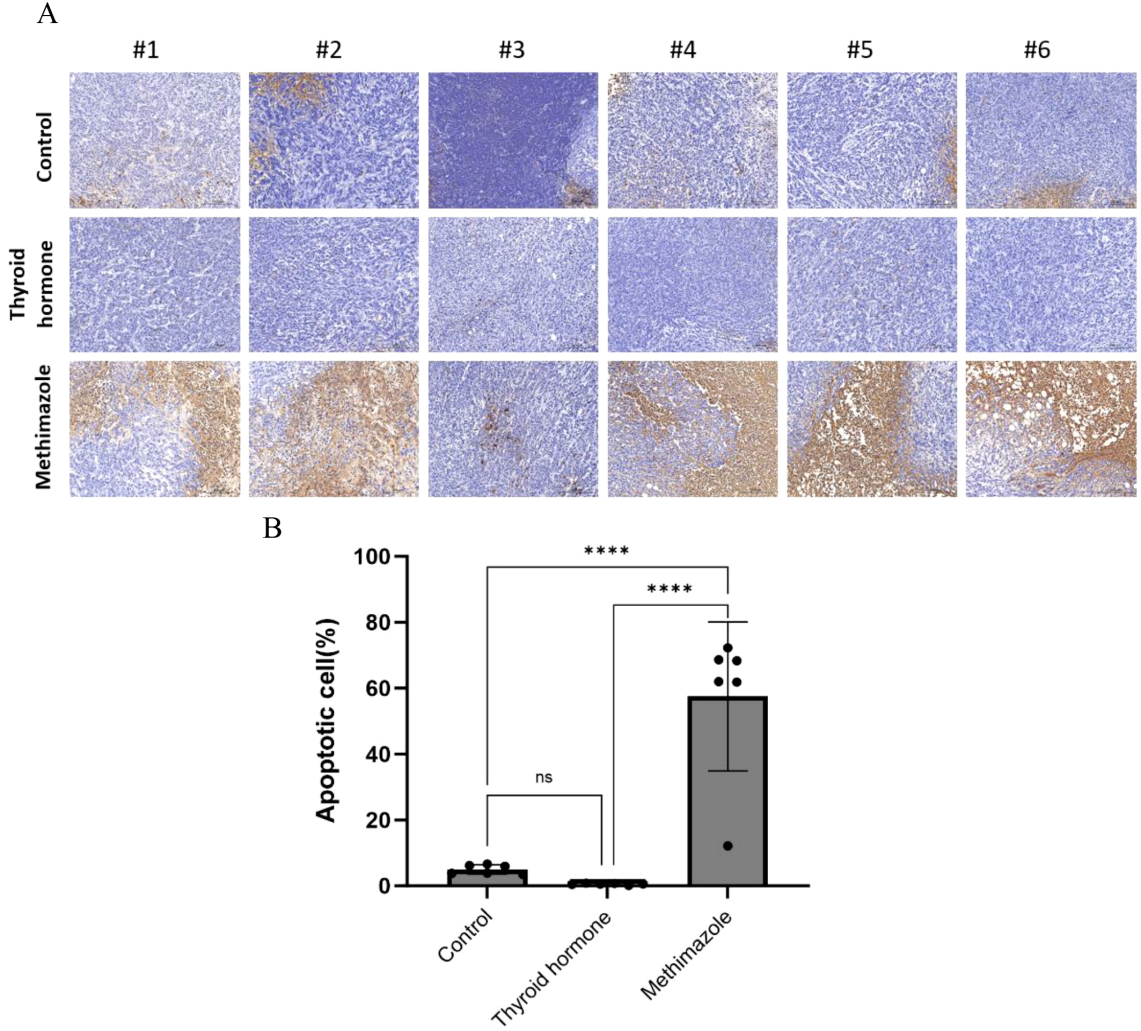
Comparison of three groups of TUNEL positive cells. **(A)** Pathological images, Scale bar:200um. **(B)** Proportion of apoptotic cells, n=6 for each group. (Data were analyzed for statistically significant differences using one-way. ns, no statistical significance, ****P< 0.0001).

The TUNEL staining technique is used to stain intact apoptotic nuclei or apoptotic bodies *in situ*. The blue marker serves as a representation of the nucleus, while the brown marker within the nucleus signifies TUNEL positivity, thereby indicating the presence of apoptotic cells. A visual examination of the pathological images revealed that the subcutaneous tumor tissues of the saline group and the levothyroxine group exhibited an absence of brown cells, while a significant presence of brown cells was observed in the methimazole groups (as depicted in **Figure 5A**). According to the TUNEL staining results, the proportion of TUNEL-positive cells in the levothyroxine group was marginally lower than that in the saline group. However, this difference is not statistically significant, meaning that it is not possible to draw any solid conclusions from it. (P>0.05, see **Figure 5B**). Conversely, the methimazole group exhibited a significant increase in the proportion of TUNEL-positive cells when compared to both the levothyroxine groups and the saline groups (P<0.0001, as shown in **Figure 5B**).

## Discussion

Thyroid hormones are known to exert their effects by binding on nuclear thyroid hormone receptors (TRs), which then bind to thyroid hormone response elements in the nucleus. These receptors are widely distributed across various tissues and play a crucial role in regulating the expression of target genes in different cellular contexts. The K-Ras gene is known to contribute to the inactivation of the tumor suppressor gene APC, marking a critical initial step in the development of most colorectal tumors. Recent studies have demonstrated that thyroid hormones can promote the nuclear accumulation of HMGA2 and β-catenin in colorectal cancer cells, with varying K-RAS mutation states, in a concentration-dependent manner, ultimately facilitating cancer proliferation (15–17). Research evidence also suggests that extracellular T4 and its extended T3 metabolite may initiate cancer cell proliferation across various malignancies by binding to plasma membrane integrin avβ3 receptors, which triggers the activation of ERK1/2 signaling cascades and downstream pathways (18). Furthermore, a recent investigation has revealed that programmed death ligand 1 (PD-L1) enhances the expression of the cell cycle regulator BUB1 by interacting with thyroid hormone receptor-associated protein 3 (THRAP3). This accelerates cell cycle progression and promotes cell proliferation. Colorectal cancer harboring the BRAFV600E mutation has shown an upregulation of PD-L1. Examination of the intracellular functions of PD-L1 has found that PD-L1 is highly expressed in both the cytoplasm and nucleus of BRAF-mutated colon cancer cells and tissues. This suggests that thyroid hormones may accelerate cell cycle progression and promote cell proliferation (19). Studies have shown that the reverse T_3_ (rT_3_), produced by the converting T_4_ to T_3,_ may support cancer growth (20). The intrinsic cellular signaling pathways of thyroid hormones in colon cancer are complex and interconnected. Overall, however, the above studies suggest a positive correlation between thyroid hormones and colon cancer proliferation.

To simulate mild hypothyroidism, methimazole was administered to nude mice via an intraperitoneal injection, resulting in a significant reduction in thyroid hormone levels. The efficacy of this induction was subsequently validated through an ELISA experiment. The final results revealed that tumor growth in the methimazole group was slower, with tumor weight showing a significant inhibitory trend compared to the control group. These results suggest that reduced thyroid hormone levels may inhibit tumor growth in the early stages of colon cancer. Subsequent pathological analysis of subcutaneous tumors revealed that hematoxylin and eosin (HE) staining showed loose tumor tissue structure in the methimazole group, with disrupted cell morphology and evident disintegration. These observations indicate significant pathological changes in the subcutaneous tumor tissue of the methimazole group. Furthermore, immunofluorescence staining showed that Ki67 expression was lower in the methimazole group than in the saline group, and much lower than in the levothyroxine group. This underscores the inhibitory effect of reduced thyroid hormone levels on tumor proliferation. Furthermore, the TUNEL staining experiment revealed a significant increase in the proportion of TUNEL-positive cells in the subcutaneous tumor tissue of the methimazole group, indicating that reduced thyroid hormone levels may promote increased apoptosis rates in these tumor cells.

To simulate mild hyperthyroidism, levothyroxine sodium was injected intraperitoneally into nude mice, thereby increasing thyroid hormone levels. Tumor growth in the thyroid hormone group was faster than in the control group. However, following data analysis, it was found that, compared with the control group, tumor mass, final tumor volume, relative cytoplasmic area, proportion of Ki67-positive cells and proportion of apoptotic cells were not statistically significant (P>0.05). Therefore, the conclusion that an increase in thyroid hormone concentration can accelerate the growth and reproduction of tumor cells is inconclusive and requires further research.

There are notable limitations in the experimental design that should be addressed in future studies. Firstly, it should be noted that the present experiment only focuses on the impact of fluctuations in single thyroid hormone concentrations on colon cancer. The gradient concentrations of thyroid hormones and methimazole can act separately on subcutaneous tumor-bearing nude mice, and the differences between the groups can be analyzed further. Secondly, prior to euthanizing the nude mice, blood samples obtained in this experiment were subjected to ELISA. The concentrations of T3, T4 and TSH obtained from these blood samples only reflect the thyroid hormone concentration before the mice died. Consequently, analyzing multiple blood samples obtained at various phases of the nude mice’s growth cycle provides a more comprehensive and precise reflection of fluctuations in thyroid hormone concentration. At the same time, a caspase 3/7 activity assay can be used to supplement the TUNEL assay. Thirdly, this experiment provides descriptive results only; further exploration is required into the impact of thyroid hormone concentration fluctuations at the molecular level of colorectal cancer. It is important to note that this study only examined the impact of thyroid hormones on colon cancer. The potential ramifications of these findings for other tumor cells, particularly the possibility of a similar effect resulting from lowered thyroid hormone levels in the body, remain to be explored.

In summary, there is a clear and significant correlation between fluctuations in thyroid hormone levels and the proliferation and growth of colorectal cancer. Research has shown that reducing thyroid hormone levels slightly can effectively inhibit the growth of colorectal cancer cells, induce apoptosis and provide new ways to treat colorectal cancer. For example, in cases of newly diagnosed colon cancer, anti-thyroid hormone medication can be used to reduce the growth and proliferation of cancer cells and accelerate tumor cell death. For patients with advanced colorectal cancer, administering anti-thyroid hormone drugs alongside chemotherapy drugs is a therapeutic strategy that can facilitate surgical intervention. Furthermore, the experiment demonstrated the harmful effects of thyroid hormones on the body. Therefore, patients diagnosed with hyperthyroidism must closely monitor their thyroid hormone levels to prevent the onset and progression of colorectal cancer. These findings may be valuable in educating patients with hyperthyroidism about their condition.

## Data Availability

The raw data supporting the conclusions of this article will be made available by the authors, without undue reservation.
