# Supplementary material for: The impact of thyroid hormone concentration fluctuations on colon cancer proliferation and growth
Source: Front Endocrinol (Lausanne). 2025 Jul 17;16:1576665. doi: 10.3389/fendo.2025.1576665 (PMC12310445; doi:10.3389/fendo.2025.1576665)
Supplement: Supplementary file 1 [file DataSheet1.docx]

Supplementary Material

# Supplementary Data
